# Indigenous Slaughter Techniques: Effects on Meat Physico-Chemical Characteristics of Nguni Goats

**DOI:** 10.3390/ani11030858

**Published:** 2021-03-18

**Authors:** Zwelethu Mfanafuthi Mdletshe, Michael Chimonyo, Cletos Mapiye

**Affiliations:** 1 Animal and Poultry Science, School of Agricultural, Earth and Environmental Sciences, University of KwaZulu-Natal, Private Bag X01, Pietermaritzburg 3209, South Africa; zwelethu.mdletshe@ul.ac.za; 2 Department of Agricultural Economics and Animal Production, Faculty of Science and Agriculture, University of Limpopo, Private Bag X1106, Sovenga 0727, South Africa; 3 Department of Animal Sciences, Faculty of AgriSciences, Stellenbosch University, Private Bag X1, Matieland 7602, South Africa; cmapiye@sun.ac.za

**Keywords:** indigenous slaughter method, Nguni, chest floor piercing, suprasternal notch piercing, transverse neck incision, ultimate meat pH

## Abstract

**Simple Summary:**

In smallholder communal farming systems, Nguni people slaughter goats using indigenous methods which have cultural and spiritual significance to them. Nguni wethers were slaughtered using the transverse neck incision (TNI), suprasternal notch (SNP) and under the shoulder blade at the chest floor point of elbow piercing (CFP) to the direction of the heart to assess meat physico-chemical characteristics parameters. Nguni wethers slaughtered with the TNI and CFP methods produced meat with an acceptable appearance similar to goats slaughtered with the conventional standard procedures.

**Abstract:**

To improve goat meat quality in rural communities, it is important to evaluate the effects of indigenous slaughter methods used by resource-limited farmers when performing traditional ceremonies on the meat physico-chemical characteristics. The current study assessed the effects of the meat physico-chemical characteristics of Nguni goats slaughtered with the transverse neck incision (TNI), suprasternal notch piercing (SNP) and the under shoulder blade piercing at the chest floor point of elbow (CFP) to the direction of the heart methods. Thirty Nguni weathers were randomly assigned to three slaughter treatments (10 goats/treatment). *Musculus longissimus thoracis et lumborum* (LTL) was sampled at post-mortem for physico-chemical characteristic measurements. Meat from wethers slaughtered with the SNP method had greater ultimate pH values than meat from wethers slaughtered with TNI and CFP slaughter methods. Wethers slaughtered with the SNP method had lower meat redness (a*), yellowness (b*), and chroma (C*) values than those slaughtered with TNI and CFP slaughter methods. Goat slaughter method did not affect meat drip loss, water holding capacity, cooking loss and shear force. Overall, Nguni wethers slaughtered with the TNI and CFP methods produced chevon with fresher meat appearance than those slaughtered using the SNP method.

## 1. Introduction

Meat is an essential source of nutrients for most people [1]. In developing countries (e.g., Africa and the Middle East) where more than 90% of the world’s goat population is found [2], goats are the second most important and abundant ruminant livestock species following cattle [3]. Such importance is due to their ability to graze and browse poor quality forage, survive feed- and water-scarce [4]. Furthermore, goats have high flock efficiency making them a short term investment [5]. Goats are owned and kept by smallholder farmers for meat (chevon), milk, manure, skin and hides [6]. Although smallholder farmers keep goats for socio-economic purposes, the primary reason is to use them for religious and cultural beliefs [7].

In 2018, FAOSTAT [2] estimated the world’s total goat population at 1 × 10^9^, with 40% of the population found in Africa. Southern Africa contribute 2% of Africa’s goat population and 50% of that are found in South Africa [8]. Half of South Africas’ smallholder households own goats, and more than 95% of goats are sold in the informal market for religious and cultural purposes [9]. When performing cultural practices, resource-limited households slaughter goats using indigenous methods [10,11], which have cultural meaning to them. The welfare of animals at commercial slaughterhouses is governed by the South African Meat Safety Act of 2000, which promotes stunning before exsanguination to ensure that animals are unconscious and experience minimal pain and suffering [11]. However, the Act accepts indigenous slaughter methods when done for personal use and cultural purposes. Indigenous slaughter methods include the transverse neck incision (TNI), suprasternal notch piercing (SNP) and the under shoulder blade piercing at the chest floor point of elbow (CFP) to the direction of the heart [12]. Cultural beliefs invoked when slaughtering goats using indigenous methods include celebrating circumcision, marriages and births, venerating ancestors, avenging evil spirits, and performing a ritual during funerals [13,14]. Immediately after slaughter and dressing, in most cases, the carcass is hanged with a metal hock in an empty room for 24 h for them to cool slowly, tenderize, dry and allow maximum blood loss. Such is important to resource-limited farmers because consuming meat with blood is prohibited, as it is believed that ancestral spirits do not accept meat with blood [13]. Offals are cleaned and consumed on the day of slaughter as they are highly perishable. Although indigenous slaughter methods have cultural meaning, therefore, approved by the Meat Safety Act of 2000, they affect the welfare and the meat physico-chemical characteristics of goats.

Several studies published the effects of slaughter methods on meat physico-chemical characteristics focused on conventional methods, halal and kosher [15,16,17], and less, if any, on indigenous slaughter techniques used by resource-limited rural households in developing countries. While studies evaluating the effects of slaughter without stunning on meat physico-chemical characteristics are available [16,17], there is a general perception that such slaughter methods have negative effects on meat quality since unconsciousness is not induced before bleeding. Understanding the effects of indigenous slaughter methods on physico-chemical attributes of goat meat will assist policymakers in developing cost-effective, humane and culturally appropriate animal slaughter regulations. Therefore, the objective of the current study was to assess the effect of TNI, SNP and CFP methods on physico-chemical characteristics of Nguni goat meat.

## 2. Materials and Methods

### 2.1. Compliance with Ethical Clearance

The Animal Ethics Committee of the University of KwaZulu-Natal (AREC/001/018D) approved the experiment following the University guide which is compliant with the South African National Standards (SANS 10386:2008) for the care and use of animals in research and teaching.

### 2.2. Goats and Experimental Design

Thirty clinically healthy Nguni wethers with an average age of 16 months old and the bodyweight of 16.8 ± 1.05 kg were purchased from the local rural farmers of Nongoma (27°53′ S, 31°38′ E) in KwaZulu-Natal Province, South Africa. Resource-limited farmers managed goats in communal rangelands dominated by *Vachellia karroo* browse species. Goats were herded during the day and housed at night. Classification of goats as Nguni breed was based on the multiple coat colour patterns, small and compact frame size [18,19]. Goats were held in lairage for 16 h before slaughter with full access to water but without feed. At slaughter, goats were assigned to three (TNI, SNP and CFP) slaughter treatments (10 goats/treatment) in a completely randomized design. The slaughter process began at 05:00 a.m. and ended at 09:00 a.m. in the morning.

### 2.3. Treatments

**Figure 1:**
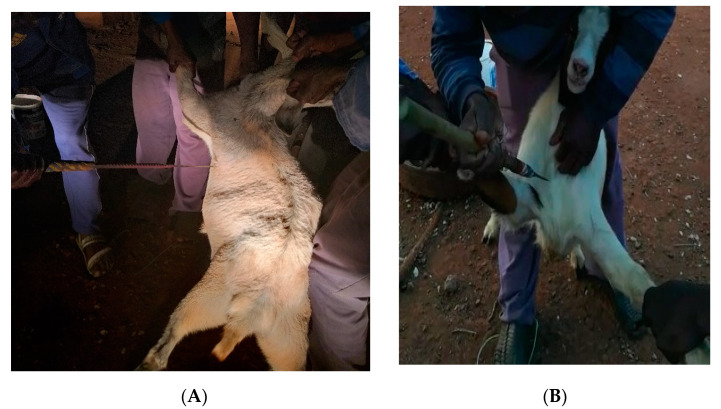
Visual pictures of the chest-floor point-of-elbow (**A**), and suprasternal notch piercing (**B**). Mdletshe et al. (2020), [12] with permission from MDPI.

Slaughter of goats followed the Meat Safety Act (2000) regulations of South Africa. Wethers in the three slaughter treatments (*n* = 10/treatment) were subjected to; transverse neck incision (TNI), under the shoulder blade at the chest floor point of elbow (CFP; Figure 1A) and suprasternal notch piercing with a short spear (SNP; Figure 1B). A sharp knife or a short spear designed explicitly for the slaughtering of goats was used. Slaughter of goats for the TNI was performed with a sharp knife. The knife severed the skin, muscles (brachiocephalic, sternocephalic, sternohyoid, and sternothyroid), trachea, oesophagus, carotid arteries, jugular veins and the major, superficial and deep nerves of the cervical region as described by Kiran [20]. Each cut was a change in the direction of movement of the knife. The SNP slaughter process was performed by two experienced slaughtermen using a short spear (Figure 1B). During slaughter, each goat was allowed to stand upright with hind legs. One slaughter man held the left front leg and the head (using horns) while the second slaughter man held the right front leg and the spear. The second slaughter men pierced the goat with the spear in the suprasternal notch in the direction of the heart. For the CFP method, piercing slaughter process was performed by three slaughtermen using a short spear (Figure 1A). Two slaughter men held each goat with front legs, allowing it to stand upright with hind legs. The third slaughter men pierced the goat on the heart girth position, next to the chest floor and point elbow to the direction of the heart. Spears and knives used during the slaughter and dressing of carcasses were sanitized by submerging them in boiling water for 1s. Blood was collected in buckets during the bleeding process.

### 2.4. Meat Sampling and Storage

The average dressed carcass weight was 8.60 ± 0.90 kg. The left and right *longissimus thoracis et lumborum* (LTL) were immediately removed between the 8th and 13th rib, immediately after dressing the carcass, vacuum-packed and placed in polystyrene cooler boxes (<4 ℃) and transported for 380 km to the Animal Science laboratory, at the University of KwaZulu-Natal, Pietermaritzburg, South Africa for physical and chemical analyses. On arrival at the laboratory after travelling for 8 h, meat was stored at room temperature (20 ℃) for 12 h pending analyses.

### 2.5. Measurements

#### 2.5.1. Meat pH

Meat pH was measured 45 min and 24-h post-mortem using a portable pH meter probe (CRISON pH25, CRISON instrument SA, Barcelona, Spain).

#### 2.5.2. Meat Colour

Meat colour was measured in triplicate 24 h after slaughter using a colour meter (HunterLab, ColorFlex EZ Spectrophotometer, Reston, VA, USA). The parameters used to evaluate meat colour followed colour CIE (1976) coordinates which measured: lightness (L*), redness (a*) and yellowness (b*) from three locations on the cut surface of individual meat samples. The Hue angle (H*) and chroma (C*) values were computed as follows:

(1) $$H^{*}=\;\tan^{-1}\left(\frac{b^{*}}{a^{*}}\right);\;C^{*}=\;\left(\sqrt{a^{*2}+b^{*2}}\right).$$

#### 2.5.3. Drip Loss and Water Holding Capacity

Drip loss was determined by suspending a standardized (40–50 g and approximately 30 × 60 × 25 mm) sample of meat in an inflated transparent plastic bag and placed in a refrigerator for 48 h at 4 °C [21,22,23]. Percentage drip loss was calculated by dividing weight loss by initial weight and multiplying by 100.

Water holding capacity (WHC) was determined by compressing approximately 3–4 g of meat with 30 kg of weight for 5 min using a texture analyser (Stable Micro System, Model TA.XT 2i/25, Godalming, UK). The water content of meat was determined by multiplying the initial weight of meat with 0.7 since the water content is 70% [24]. Water loss was determined by subtracting final weight from the initial value. Percentage water holding capacity was calculated by subtracting water loss from water content, dividing by water content and multiplying by 100.

#### 2.5.4. Cooking Loss and Shear Force

Fresh LTL samples were cut and weighed (initial weight) to form individual standardized slices of approximately 50 mm thick [25]. Meat samples were placed in a RATIONAL Granite-enamelled container 20 mm deep. A RATIONAL SCC 61E self-cooking centre (Landsberg, Munich, Germany) was used to roast LTL samples. The hot plate was preheated for 5 min to 205 ℃ [26]. Immediately after preheating, a RATIONAL Granite-enamelled container 20 mm deep was placed onto a RATIONAL Grid; stainless steel 1/1GN were meat was roasted for 4 min. After completing the cooking process, meat samples were cooled at room temperature and weighed. Percentage cooking loss was calculated as [(Initial weight of the sample—the weight of cooked sample) ÷ initial weight of meat sample × 100].

Following cooking, sub-samples of specified core diameter parallel to the grain of the meat were used to determine WBSF (Warner-Bratzler Shear Force). Samples were sheared perpendicular to the fibre direction using a texture analyser model TA.XTplus, texture analyser (Stable Micro System, Model TA.XT 2i/25, UK). The mean maximum load recorded for the three cores were represented as the average of peak force in Newton’s (N) for each sample.

### 2.6. Statistical Analyses

All data were analysed using statistical analysis software (SAS) [27]. Normality of residuals tests for physico-chemical characteristics was computed using the UNIVARIATE procedure to assess data distribution. A general linear model procedure was used to test the effects of indigenous slaughter method on meat quality parameters.

The following model was used:

(2) $$Y_{ij}=\mu +S_{i}+\varepsilon_{ij}$$

where

$Y_{ijk}$ = Response variables (pH, a*, b*, L*, H*, C*, drip loss, water holding capacity, cooking loss, and shear force; dressing percentage);

μ = population mean;

$S_{i}$ = effect of indigenous slaughter method;

$\varepsilon_{i}$ = residual error.

The significance threshold for differences between least square means was set at *p* ≤ 0.05.

## 3. Results

Ultimate pH_24 h_, redness (a*), yellowness (b*) and chroma (C*) was not normally distributed (*p* ≤ 0.05). Initial live weight, dressed weight and dressing percentage were not different comparing slaughter treatments (Table 1). Slaughter method had no significant influence on pH 45 min (Table 1). Slaughter method had a significant effect on the ultimate pH_24 h_. It was greater for the SNP when compared with the TNI and CFP slaughter methods (Table 1).

Slaughter method influenced meat redness (a*), yellowness (b*) and chroma (Table 1) values of meat from goats slaughtered using SNP method were lower than those of meat from goats slaughtered using TNI and CFP methods (Table 1). Slaughter method had no significant influence on the lightness (L*) and hue (H*) coordinates (Table 1). Similarly, no significant effect was observed for drip loss, WHC, cooking loss, shear force and dressing percentage.

## 4. Discussion

The high pH observed in meat from animals slaughtered using the SNP method is higher than Nguni goats slaughtered without stunning using the conventional methods [28,29]. This suggests these animals were more stressed than those slaughtered using other methods [16,17]. This could be explained by prolonged stress before and during slaughter, which may have led to a reduction in glycogen levels, and therefore low post-mortem lactic acid production [16,28,30]. The causes of stress are, however, not immediately apparent. The prolonged stress could be related to nature, frequency, strength, severity, intensity, and duration of the stressor(s) before and during slaughter [12]. Such stressors include animal handling before and during slaughter and pain experienced by the animal during sticking and exsanguination [31]. These stressor(s) were not measured in the current study. Stress before and during slaughter can be measured by assessing cortisol and catecholamine concentration in urine [32]. Pain experienced by goats during sticking and exsanguination can be evaluated by comparing changes in electroencephalographic (EEG) activities with possible noxious stimuli [33]. Such merit further investigation.

The lower meat redness (a*) observed for the SNP slaughter treatment could have been influenced by prolonged stress before slaughter, resulting in the depletion of muscle glycogen, decreasing the amount of substrate available for anaerobic metabolism at the time of slaughter. Such limits post-mortem glycolysis, therefore resulting in high pH_24 h_, which is a factor that promotes mitochondrial respiration and competition for oxygen in meat [34,35]. High pH_24 h_ also results in low protein denaturation, which creates a more closed tissue structure. The closed structure reduces the diffusion of oxygen into the meat from the surface, and any oxygen that does not reach the interior is then utilized by high cytochrome activity which out-competes myoglobin for oxygen encouraged by high pH [36]. As a result, less oxymyoglobin is formed, and consequently, meat looks less red.

The observation that the SNP method had lower meat yellowness (b*) values than other methods could be explained by the effect of high meat pH_24 h_ on oxygenation of the myoglobin [37,38,39]. The lower chroma (C*, colour intensity) values observed for the SNP slaughter method compared to other methods also corresponds high pH_24 h_ values, which negatively correlates with low oxymyoglobin content in meat [40,41]. Such is influenced by the interaction between histidine from and bound oxygen which repel iron atoms out of the plane of porphyrin, making oxymyoglobin less compact. It is a well-known phenomenon that a decrease in oxymyoglobin content in meat is accompanied by lower values of a*, b* and C* [42,43].

The finding that slaughter method did not affect drip loss, WHC, cooking loss and shear force agree with earlier reports [17,44,45]. Overall, the high pH (6.4–6.8), WHC, and low drip loss, a*, b*, and chroma values reported across slaughter methods are a characteristic of a dark firm and dry (DFD) meat [46,47]. This implies that all the goats across treatments had prolonged stress before and/or during slaughter and that could be related to the high temperamental behaviour of the Nguni goat breed among other stress factors [48]. Further studies to determine the pre-, peri- and post-mortem glycogen reserves, lactic acid concentration in muscles and stress hormones in Nguni goats could be necessary for explaining the effect of their temperamental behaviour on meat quality. Causes of pre- and perimortem stress that could be significant in minimising DFD meat from Nguni goats also merit investigation.

## 5. Conclusions

Nguni goats slaughtered using SNP slaughter method had higher pH_24 h_ than other slaughter methods, which resulted in lower meat redness, yellowness and chroma values. It was concluded that Nguni goats slaughtered using TNI and CFP methods may produce chevon with better meat colour than those slaughtered with the SNP method. The high meat pH_24 h_, WHC, and low drip loss and colour (a*, b*, and chroma) indicate DFD meat. Further studies to determine the causes of DFD meat in Nguni goats slaughtered using indigenous methods are important.

## Figures and Tables

**Table 1:** Effects of indigenous slaughter methods on physico-chemical attributes of goat meat.

Variable	Slaughter Method			Significance
	TNI	SNP	CFP	
Intial weight	17.3 ± 0.31	16.5 ± 0.42	16.1 ± 0.38	NS
Dressed weight	8.44 ± 0.31	8.80 ± 0.42	8.67 ± 0.38	NS
pH_45 min_	7.79 ± 0.10	7.69 ± 0.10	7.58 ± 0.10	NS
pH_24 h_	6.40 ± 0.13 ^b^	6.82 ± 0.13 ^a^	6.42 ± 0.13 ^b^	*
Drip loss (%)	3.0 ± 0.75	1.75 ± 0.87	1.88 ± 0.75	NS
Water holding capacity (%)	74.2 ± 1.28	75.6 ± 2.21	72.9 ± 1.57	NS
Colour parameters				
a*	16.4 ± 0.56 ^a^	14.7 ± 0.56 ^b^	16.3 ± 0.56 ^a^	*
b*	13.5 ± 0.61 ^a^	11.5 ± 0.61 ^b^	13.2 ± 0.61 ^a^	*
L*	29.5 ± 1.02	28.3 ± 1.02	30.6 ± 1.02	NS
H*	0.69 ± 0.01	0.66 ± 0.01	0.68 ± 0.01	NS
C*	21.2 ± 0.78 ^a^	18.7 ± 0.78 ^b^	21.0 ± 0.78 ^a^	*
Cooking loss (%)	22.7 ± 2.3	21.6 ± 3.0	19.4 ± 2.6	NS
Shear force (N)	9.52 ± 0.72	10.7 ± 0.83	10.6 ± 0.72	NS
Dressing (%)	48.8 ± 1.74	53.4 ± 2.34	53.9 ± 2.13	NS

^a,b^ Means in the same row with different superscripts are significantly different at * *p* ≤ 0.05; NS: not significant; TNI: transverse neck incision; SNP: suprasternal notch piercing; CFP: chest floor piercing; pH_45min_: pH 45 min post-mortem; pH_24 h_: ultimate pH; L*: lightness; a*: redness; b*: yellowness; H*: Hue; C*: chroma.

## Data Availability

The data presented in this study is available on request from the corresponding author. The data is not publicly available because participants did not consent.

## Abbreviations

TNI	transverse neck incision
SNP	suprasternal notch piercing
CFP	under shoulder blade chest floor point of elbow piercing
LTL	muscle longissimus thoracis et lumborum
*a**	Redness
*b**	Yellowness
*C**	chroma values
FAOSTAT	Food and Agriculture Organization Corporate Statistical
L	litre
WHC	Water holding capacity
GLM	general linear model
SAS	statistical analysis software
PDIFF	Requests that *p*-values for differences
DFD	dark: firm and dry
